# Characterization of the Functional Dynamics in the Neonatal Brain during REM and NREM Sleep States by means of Microstate Analysis

**DOI:** 10.1007/s10548-021-00861-1

**Published:** 2021-07-13

**Authors:** Mohammad Khazaei, Khadijeh Raeisi, Pierpaolo Croce, Gabriella Tamburro, Anton Tokariev, Sampsa Vanhatalo, Filippo Zappasodi, Silvia Comani

**Affiliations:** 1 Department of Neuroscience, Imaging and Clinical Sciences, University “Gabriele d’Annunzio” of Chieti–Pescara, Chieti, Italy; 2 Behavioral Imaging and Neural Dynamics Center, University “Gabriele d’Annunzio” of Chieti–Pescara, Chieti, Italy; 3 Department of Clinical Neurophysiology, BABA Center, Pediatric Research Center, Children’s Hospital, Helsinki University Hospital and University of Helsinki, Helsinki, Finland; 4 Neuroscience Center, Helsinki Institute of Life Science, University of Helsinki, Helsinki, Finland; 5 Institute for Advanced Biomedical Technologies, University “Gabriele d’Annunzio” of Chieti–Pescara, Chieti, Italy

**Keywords:** Neonatal EEG, EEG microstate analysis, Sleep states, Neonatal brain dynamics

## Abstract

Neonates spend most of their life sleeping. During sleep, their brain experiences fast changes in its functional organization. Microstate analysis permits to capture the rapid dynamical changes occurring in the functional organization of the brain by representing the changing spatio-temporal features of the electroencephalogram (EEG) as a sequence of short-lasting scalp topographies—the microstates. In this study, we modeled the ongoing neonatal EEG into sequences of a limited number of microstates and investigated whether the extracted microstate features are altered in REM and NREM sleep (usually known as active and quiet sleep states—AS and QS—in the newborn) and depend on the EEG frequency band. 19-channel EEG recordings from 60 full-term healthy infants were analyzed using a modified version of the k-means clustering algorithm. The results show that ~ 70% of the variance in the datasets can be described using 7 dominant microstate templates. The mean duration and mean occurrence of the dominant microstates were significantly different in the two sleep states. Microstate syntax analysis demonstrated that the microstate sequences characterizing AS and QS had specific non-casual structures that differed in the two sleep states. Microstate analysis of the neonatal EEG in specific frequency bands showed a clear dependence of the explained variance on frequency. Overall, our findings demonstrate that (1) the spatio-temporal dynamics of the neonatal EEG can be described by non-casual sequences of a limited number of microstate templates; (2) the brain dynamics described by these microstate templates depends on frequency; (3) the features of the microstate sequences can well differentiate the physiological conditions characterizing AS and QS.

**Supplementary Information:**

The online version contains supplementary material available at 10.1007/s10548-021-00861-1.

## Introduction

Sleep plays a crucial role in the development of cortical pathways and brain networks in the neonate. These phenomena, which set up the foundation of future behavior and memory, are formed by endogenous driven brain activity during neonatal sleep (Koolen et al. 2014; Lubsen et al. 2011; Omidvarnia et al. 2014). Falling asleep and sleep state transitions are events that involve the reorganization of functional interactions between remote brain regions (Tokariev et al. 2019a; b). Such long-range neural connections are a key component in early brain functional development. Hence, understanding the brain networks underpinning the different sleep states in neonates is of great importance to achieve insights into neurological well-being (Bennet et al. 2018). However, little is known about the mechanisms underlying the functional communication within the neonatal brain and advancing our knowledge of the dynamic reorganization of the functional networks underpinning sleep states in the neonatal brain can contribute to assess the normal neurodevelopment and to improve the early diagnosis and prediction of neurodevelopmental disorders.

Electroencephalography (EEG) is a non-invasive and accurate tool that has been widely used for detecting and evaluating large-scale spatial coordination in the electrical activity of the neonatal brain (Pedersen et al. 2017; Tokariev et al. 2012, 2019a). The functional interaction between different brain areas can be assessed through different methods, the most common one being pairwise connectivity analysis of EEG signals in a specific time window and for specific frequency bands. Several studies on the adult EEG have demonstrated that brain dynamics can be modeled by means of a sequence of transient, non-overlapping patterns of quasi-stable electrical potentials named “microstates” (Khanna et al. 2015; Michel and Koenig 2018). Microstates capture the broad-band brain dynamics that result from the functional interactions of widespread ensembles of neural sub-units organized in a hierarchical architecture (Michel and Koenig 2018). Cluster analysis of microstates can parse the EEG data to a non-casual sequence of short-lasting classes of brain electrical states during which distributed neural sources are synchronously active and generate stable potential topographies on the scalp. A low number of microstates was demonstrated to comprise a high proportion of the ongoing broad-band EEG activity in the adult brain, hence enabling to represent the global brain dynamics associated with a given condition, such as resting state, with a specific sequence of microstates (Michel and Koenig 2018; Pascual-Marqui et al. 1995). Differently from other EEG analysis techniques, that evaluate brain activity at specific electrode locations, during specific time intervals and within given frequency bands, microstate analysis of EEG signals provides a global perspective on the activity of the whole cortex and an informative framework that permits to characterize the global brain activity and brain dynamics associated with sleep states in neonates through specific microstate sequences without any a priori hypothesis (Murray et al. 2008; Michel and Koenig 2018).

EEG microstates have already been used to analyze the brain dynamics during sleep in adults. Cantero et al. (1999) showed that transitions between different arousal states are associated with changes of the microstates in the alpha activity of the brain. Brodbeck et al. (2012) analyzed the microstate templates during different phases of the non-rapid-eye-movement (NREM) sleep state and showed that they had a relatively high degree of spatial correlation with those extracted during wakefulness. In another study, Xu et al. (2020) investigated the relationship between fMRI fluctuations and microstates during slow wave sleep, revealing a correlation between EEG microstates and brain functional networks. Recently, Bréchet et al. (2020) compared microstates during NREM sleep with microstates in wakefulness, showing that two microstates dominated sleep, with a different spectral content with respect to the microstates dominating wakefulness. Bréchet and colleagues also highlighted the possibility to characterize functional states of the sleeping brain, such as dreaming experiences, by means of specific dominant microstates.

However, in our knowledge the investigation of the brain dynamics during neonatal sleep using microstate analysis has not been performed so far. Neonatal sleep is characterized by the occurrence of two vigilance states: REM sleep (usually known as “active sleep” (AS) in the newborn) and NREM sleep (usually known as “quiet sleep” (QS) in the newborn) (Grigg-Damberger 2016). These states differ for the electrophysiological activity of the brain and for other physiological signs (André et al. 2010). Studies on the functional organization of the neonatal brain during sleep have focused on the functional interactions across brain areas that characterize the transitions from QS to AS on long time scales (typically several seconds) (Tokariev et al. 2012, 2016, 2019a; b; González et al. 2011; Tóth et al. 2017) and for specific frequency bands (Vanhatalo and Kaila 2006; Tokariev et al. 2012, 2016). With this approach, the rapid changes occurring in the global brain activity on much smaller time scales (typically milliseconds) are disregarded. Conversely, microstate analysis could be an effective method to detect and model the rapid dynamical changes occurring in the functional organization of the neonatal brain during AS and QS.

Based on these premises, the aims of this study were: (1) to demonstrate that the spatio-temporal dynamics of the co-activated brain areas arising in full-term neonates during QS and AS could be modeled by non-casual sequences of a limited number of microstates; (2) to verify whether the features of the neonatal microstates depend on specific frequency bands; (3) to characterize the unique microstate sequences describing the neonatal brain activity and brain dynamics during AS and QS by means of global microstate metrics.

## Materials and Methods

### Subjects and Recordings

A datasets of 60 full-term healthy newborn infants was collated from cohorts that were published earlier for other purposes (Tokariev et al. 2019a,b). In short, average gestational age (GA) at birth was 40.4 ± 1.8 weeks, and the EEG recordings were performed at an average GA of 41.3 ± 2 weeks at the Helsinki University Central Hospital.

The study design and procedures have been approved by the Ethics Committee of the Helsinki University Central Hospital (Finland). Informed written consent was received from a guardian before inclusion of an infant into the study.

The EEG was recorded using the NicOne EEG amplifier (Cardinal Healthcare/Natus, USA) and EEG caps mounting either 19 or 28 channels (sintered Ag/AgCl electrodes; Waveguard, ANT-Neuro, Germany) in a layout based on the international 10–20 system for electrode placement (Jurcak et al. 2007). The sampling frequency was not uniform for all recordings: the EEG signals were registered with a sampling frequency of 250, 256, or 500 Hz.

During the recording session, each neonate underwent both AS and QS, which were assessed by an expert using a combination of electrophysiological and behavioral measures derived from polygraphic channels, which included the electrocardiogram (ECG), the electrooculogram (EOG), the respiratory signal, and the chin electromyogram (EMG). For each neonate, the EEG recordings were split into epochs containing either AS or QS states, and then grouped according to the sleep state. The duration of the final retained epochs ranged from 120 to 300 s among all neonates. The same 19 EEG channels (Fp1, Fp2, F3, F4, F7, F8, Fz, T7, T8, C3, C4, Cz, P3, P4, P7, P8, Pz, O1, O2) were selected from all recordings for further analysis.

### Data Preprocessing

The EEG epochs were band-pass filtered with cut-off frequencies at 0.15 and 45 Hz. For this purpose, we applied a combination of seventh-order low-pass and high-pass non-causal Butterworth filters in both forward and backward directions. Then, all EEG epochs were down-sampled to 100 Hz and re-referenced to the common-average montage. The segments showing more than half of the electrodes affected by excessive noise were visually identified and clipped from the epochs. Independent Component Analysis (ICA) was applied to the EEG epochs to remove the signal components containing cardiac, ocular, myographic and respiratory artifacts (Jung et al. 2000); the retained independent components were then re-projected onto the scalp to reconstruct artefact-free EEG signals.

### Microstate Analysis

**Fig. 1:**
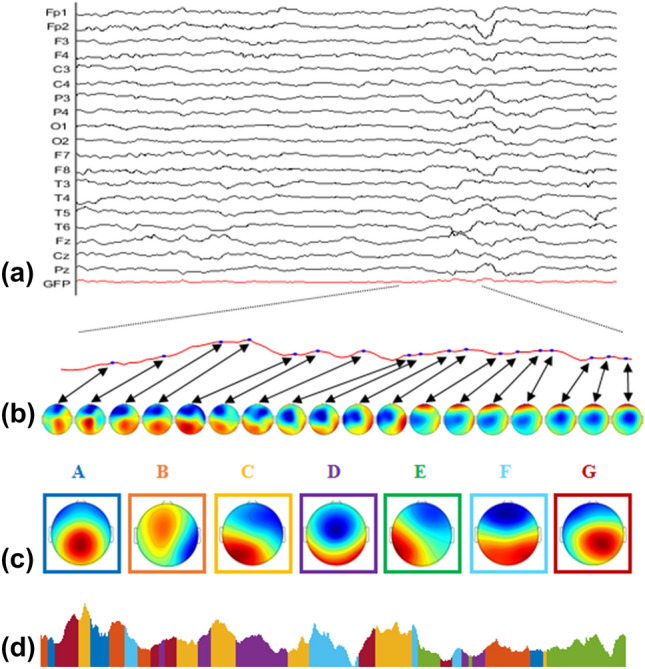
Schematic illustration of microstate analysis for the identification of the global microstates. **a** Ten seconds of spontaneous EEG recordings during QS state (neonate ID:1) against average reference (black traces) and GFP (red trace). **b** Two seconds of GFP and topographies related to the peaks of it. **c** The grand average global microstate templates labeled A to G. **d** Two seconds of microstate sequence derived by backfitting the global microstates to the GFP peaks. The color-coded areas under the curve indicate the assignment of the microstate map with the same color to the spontaneous EEG

Microstate analysis aims at identifying quasi-stable distributions of scalp electric potentials originating from brain activity (Fig. 1). This analysis is broken-down in three steps: (1) identification of the dominant microstate templates from the different distributions of the electric scalp potential; (2) representation of the EEG time course by a sequence of dominant microstates; (3) characterization of the microstate sequences—hence the represented brain dynamics—by means of global microstate metrics. The EEGLAB plugin was used to extract microstate templates and to compute the microstate metrics (www.thomaskoenig.ch/index.php/software/).

### Identification of the Dominant Microstate Templates

A two-step clustering analysis was performed using a modified version of the k-means clustering algorithm (Pascual-Marqui et al. 1995). This procedure was used to identify the dominant microstate templates of AS and QS; therefore, it was separately applied to the groups of artefact-free AS or QS epochs. The first clustering step was applied to individual EEG epochs. The global field power (GFP), defined as the standard deviation of the EEG signals across all electrodes (Lehmann and Skrandies 1980), was calculated according to Eq. (1):

1 $$GFP = ~\sqrt {\frac{{\mathop \sum \nolimits_{{j = 1}}^{C} \left( {v_{j} - \bar{v}} \right)^{2} }}{C}}$$

where $C$ is the number of channels, *v*_*j*_ is the voltage in the $jth$ channel, and $\stackrel{-}{v}$ is the average voltage across all channels at each time sample. Then, the local GFP peaks, which correspond to the time samples with the highest topographic signal to noise ratio (SNR) (Khanna et al. 2015), were identified. The scalp potential distributions corresponding to the extracted peaks were fed to the clustering algorithm, ignoring polarity inversion, to identify the dominant microstate templates (Fig. 1b). The number of possible microstate templates in the clustering algorithm was varied between 3 and 15 and the optimal number of microstate templates was chosen based on the Krzanowski-Lai (KL) criterion as the number of microstate templates corresponding to the second KL maximum value (Murray et al. 2008). To find the most representative microstate templates across all subjects within a group (i.e., the global microstate templates across all EEG epochs of the AS or QS groups), the dominant microstate templates extracted at the individual level were fed into the group-clustering spatial k-means algorithm and the sleep state-specific global microstate templates for the AS and QS states were identified.

### Reconstruction of the Dominant Microstate Sequences

To represent the brain dynamics by sequences of global microstates, the dominant microstate templates were back-fitted to the EEG signals. This was done by calculating the spatial correlation between each global template and the scalp potential distributions at each GFP peak, ignoring polarity inversion. A specific global microstate template was then assigned to each GFP peak based on the highest spatial correlation value. The time points between GFP peaks were labeled by means of linear interpolation.

### Calculation of Microstate Metrics

Individual sequences of dominant microstates can be typified by means of coded global metrics (Lehmann et al. 1987):

- Mean microstate duration (ms): the average time during which a microstate remains stable; this metric is an index of stability of the underlying brain dynamics.
- Mean microstate occurrence (Hz): average number of times per second that this microstate becomes dominant during the EEG time course; this metric indicates the tendency of underlying neural generators to be activated and become dominant.
- Mean microstate coverage (%): the percentage of the total time covered by a specific global microstate; this metric indicates the relative predominance of the activation of the neural network underlying a given microstate template with respect to the others.

In addition to the above-mentioned metrics, we evaluated whether the sequence of dominant microstates followed a specific pattern that could characterize the underlying brain dynamics. For this purpose, we estimated the *microstate syntax*, i.e. the rules governing the transition from one microstate to another during the EEG time course (Lehmann et al. 2005). Briefly, the null hypothesis states that the transition from the current global microstate to another one is independent of the current state and the transition occurs randomly. To test this hypothesis, for each pair of microstates X and Y, the *transition probability* (${P}_{X\to Y}$), i.e. the number of *observed* transitions from microstate X to Y divided by the total number of transitions among all microstates, was calculated. Under the null hypothesis (i.e., the casual transition from X to Y), the transition probability is proportional to the relative occurrence of microstates X and Y. In this case, the *expected probability* of transition from microstate X to Y is defined as:

2 $$P_{{X \to Y}}^{*} = P_{X} P_{Y} /\left( {1 - P_{X} } \right)$$

where ${P}_{X}$ (${P}_{Y}$) is the relative occurrence of microstate $X$ ($Y$) which indicates the ratio of the number of occurrences of microstate X (Y) to the total number of microstates observed. To test the differences between *expected* and *observed* transition probabilities, these probabilities were computed for each epoch. Then the differences between the mean observed transition percentage (${P}_{X\to Y}$) and the expected transition probability (${P}_{X\to Y}^{*}$) between two given dominant microstates were quantified by means of the chi-square distance (D):

3 $$D_{{X,Y}} = \mathop \sum \limits_{{X,Y}} \left( {P_{{X \to Y}} - P_{{X \to Y}}^{*} } \right)^{2} /P_{{X \to Y}}^{*}$$

where the summation is taken over all possible pairs of dominant microstates. If the transition between two dominant microstates does not depend on the current state, the *expected* transition does not differ from the *observed* transition, and the chi-square distance is equal to zero. In this case, the transition probability depends only on the occurrence of microstates and not on their sequence, as assessed by the expected probability calculated in Eq. (2). On the other hand, if the transition between two global microstate templates is driven by a law (i.e., in the case the null hypothesis is not verified), there is a structure in the microstate transitions and the chi-square distance is greater than zero. To test the statistical significance of the chi-square distance (effect size), a randomization test with 5000 repetitions was used. In this test, it was assumed that the effect size is obtained by chance (null hypothesis). First, the labels *expected* and *observed* were randomly assigned to the transition probabilities which were calculated for each group (AS and QS). Afterwards, the chi-square distance between *observed* and *expected* transition probabilities was computed. Using this procedure, the distribution of randomly generated chi-square distances was obtained. To estimate the probability associated with an effect size obtained by chance, we computed the ratio between the number of random effect sizes greater than the observed effect sizes and the total number of random effect sizes (5000). This led to a final p value stating the probability of the observed difference to be in the same distribution of the random distances.

Finally, we calculated the *directional predominance* (Lehmann et al. 2005), that quantifies, for all possible pairs of dominant microstates, the directional asymmetries in the transitions between two microstates. The directional predominance of $X\leftrightarrow Y$ is calculated as the difference between the observed transition probability to transit from X to Y and the probability to transit from Y to X. A significant positive value of $X\leftrightarrow Y$ shows a higher tendency to transit from $X$ to $Y$, while a significant negative value indicates the opposite.

### Topographical Analysis

Separately for AS and QS, we quantified the global EEG signal variance explained by the obtained sets of dominant microstate templates (Global Explained Variance—GEV). This metric quantifies the ability of the dominant microstate templates to describe the dataset (Murray et al. 2008).

Then, we evaluated the similarity of the global microstate templates of the same type in the two groups (AS and QS) by means of the topographical analysis of variance (TANOVA (Michel et al. 2009)). TANOVA is based on the evaluation of effect size between groups. We quantified the effect size by computing the global dissimilarity (GD) between pairs of global microstate templates as:

4 $$GD_{{u,v}} = \sqrt {\frac{1}{N}\mathop \sum \limits_{{i = 1}}^{N} \left( {\frac{{u_{i} }}{{GFP_{u} }} - \frac{{v_{i} }}{{GFP_{v} }}} \right)^{2} }$$

where ${u}_{i}$ and ${v}_{i}$ are the electric potentials of the ${i}_{th}$ electrode in the microstate templates $u$ and $v$ respectively; ${GFP}_{u}$ and ${GFP}_{v}$ are the global field powers of the microstate templates (*u* and *v*); *N* is the number of electrodes. ${GD}_{u,v}$ has an indirect relationship with the spatial correlation between two maps. In other words, the lower global dissimilarity is, the higher spatial correlation is.

### Narrow-Band Analysis

Based on the knowledge that the infant broadband EEG activity derives from several functional mechanisms characterized by different frequency components, we tested whether the results of microstate analysis depended on frequency. Although the power spectral density (PSD) of the neonatal EEG has a 1/f shape (see Fig. S1 in Online Resource 1 and Table S1 in Online Resource 2), we then performed microstate analysis of the EEG signals filtered into five different frequency bands: delta (0.5–4 Hz), theta (4–8 Hz), alpha (8–13 Hz), beta (13–25 Hz), and gamma (25–45 Hz). To filter the EEG signals, a combination of seventh-order low-pass and high-pass non-causal Butterworth filters in both forward and backward directions was applied. For each filtered dataset, the clustering procedure described above was applied to extract the dominant microstate templates. The microstate templates were back-fitted to the band-passed EEG signals and the GEV and other microstate metrics (duration, occurrence, and coverage) were calculated. To verify a possible linear relationship between microstate metrics and frequency, the regression line was fitted between the log-transformed values of microstate metrics (averaged across the seven dominant microstate templates) and the log-transformed median frequency of the considered five frequency bands (i.e., the frequencies 2.25, 6, 10.5, 19 and 35 Hz respectively for delta, theta, alpha, beta, and gamma bands).

### Statistical Analysis

The statistical differences between microstate metrics (duration, occurrence, and coverage) in the two sleep states (AS, QS) were assessed with a two-way repeated-measure ANOVA design with *sleep states* and *microstate templates* as within-subject factors. When the sphericity assumption was not met, the Greenhouse–Geisser correction was applied. When an interaction between *sleep states* and *microstate templates* was identified, a Bonferroni corrected post-hoc paired t-test was performed to compare the microstate metrics obtained for the two sleep states.

Similarly, the repeated-measure ANOVA was used to test the statistical significance of differences in the directional predominance between sleep states. In this case, *sleep states* and *directional predominance* were considered as within-subject factors. Post-hoc false discovery rate (FDR) (Benjamini and Hochberg 1995) corrected comparisons were performed between sleep states. To test if a significant directional predominance in the transition between two microstate templates occurred, for AS and QS separately the directional predominance values were compared with the null value employing the FDR corrected one-sample t-test.

## Results

### Optimal Number of Microstate Templates and their Spatial Configuration

**Fig. 2:**
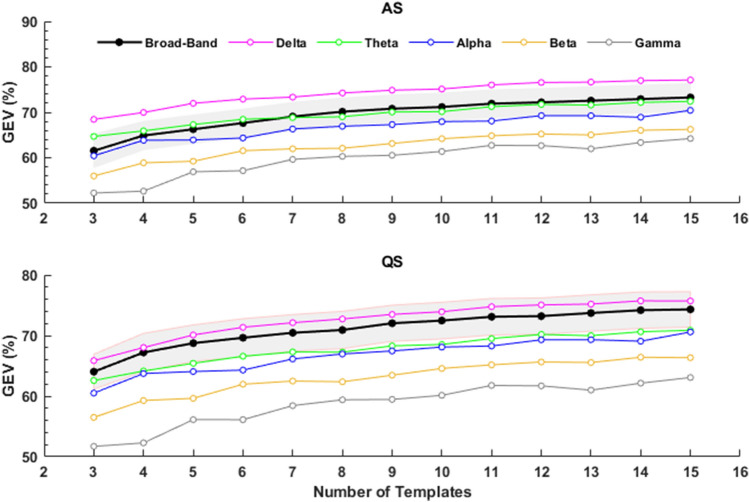
Mean values of the GEV as a function of the number of microstate templates in different frequency bands. (Broad-Band: black, Delta: magenta, Theta: green, Alpha: blue, Beta: yellow, Gamma: gray) for AS and QS. The shaded area shows the GEV standard deviation for the broad-band analysis

We observed that the GEV increased with the number of microstate templates for both AS and QS (Fig. 2, black lines). However, GEV did not increase more than 1% when the number of microstate templates varied from 7 to 15. According to the KL criterion, the optimal number of microstate templates was then equal to 7 for both sleep states. By using seven microstate templates, we obtained GEV = 69.06% ± 3.12% for the AS group and GEV = 70.45% ± 3.01% for the QS group. We also observed that GEV depended on frequency for both sleep states (Fig. 2). The GEV of the 7 global microstate templates calculated for the EEG data filtered in delta band was higher than the broad-band GEV values for both AS and QS (paired sample t test, p > 0.001 for both AS and QS), whereas the GEV of the 7 global microstate templates calculated for the alpha, beta and gamma frequency bands were always lower than the broad-band GEV values for both AS and QS (Fig. 2, p > 0.001).

**Fig. 3:**
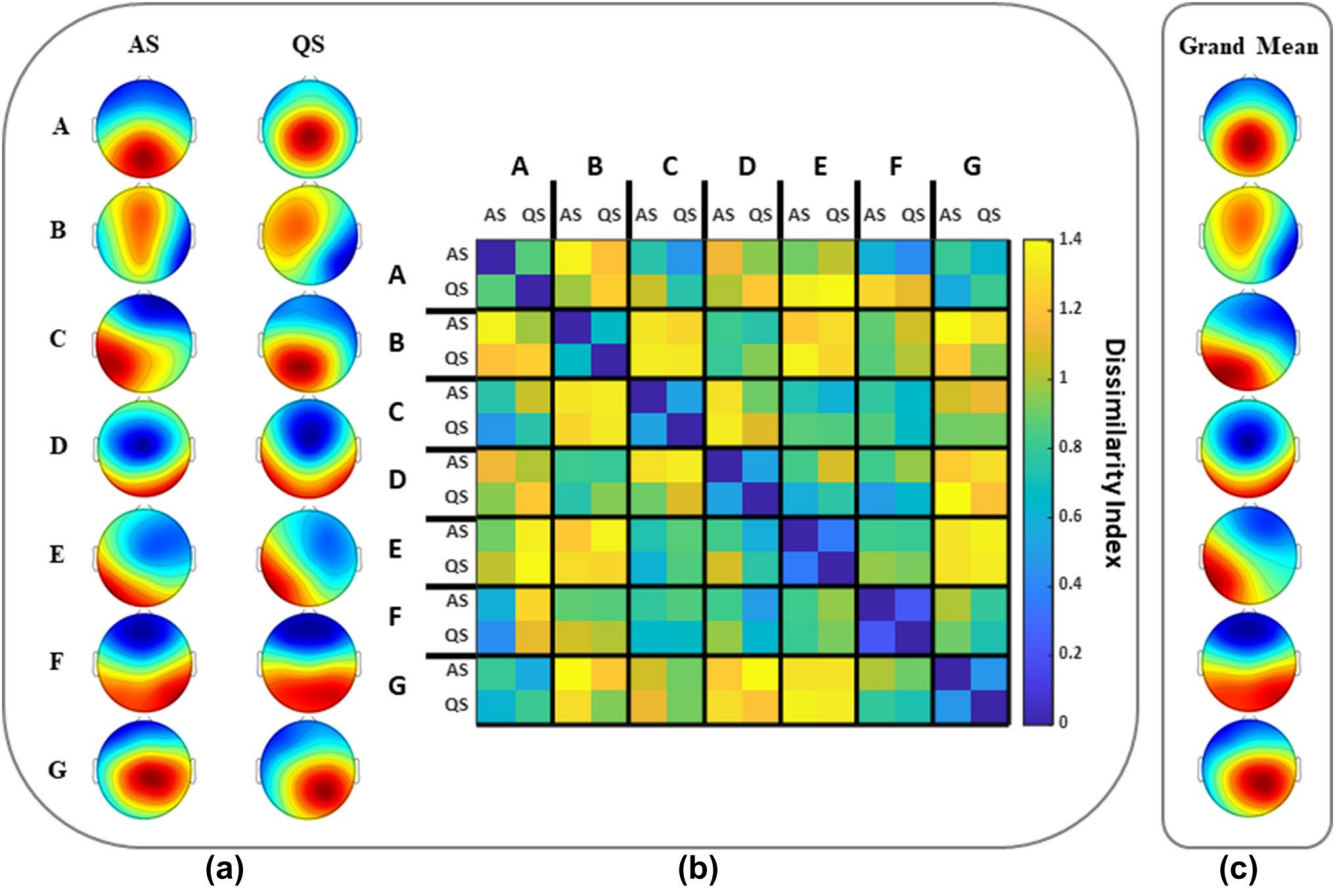
**a** The seven dominant microstate templates for AS and QS. **b** The dissimilarity matrix showing the dissimilarity index between all possible pairs of templates. **c** The grand average dominant microstate templates

The topographic comparison of the dominant microstate templates (A-G) obtained for AS and QS in broad-band data showed no significant differences between the individual templates in the two groups (p > 0.5) (Fig. 3). Therefore, subsequent analyses were performed using dominant microstate templates extracted from EEG signals pooled from both the AS and QS groups to create dominant microstate templates that were independent of the sleep state. These sleep-state-independent dominant maps were calculated by means of a cluster analysis across the AS and QS groups of all individual templates. This allowed us to directly compare the results of microstate analysis obtained for AS and QS. The GEV of these new dominant broad-band microstate templates was 68.94% ± 3.01% (Fig. 3c).

### Microstate Metrics

**Fig. 4:**
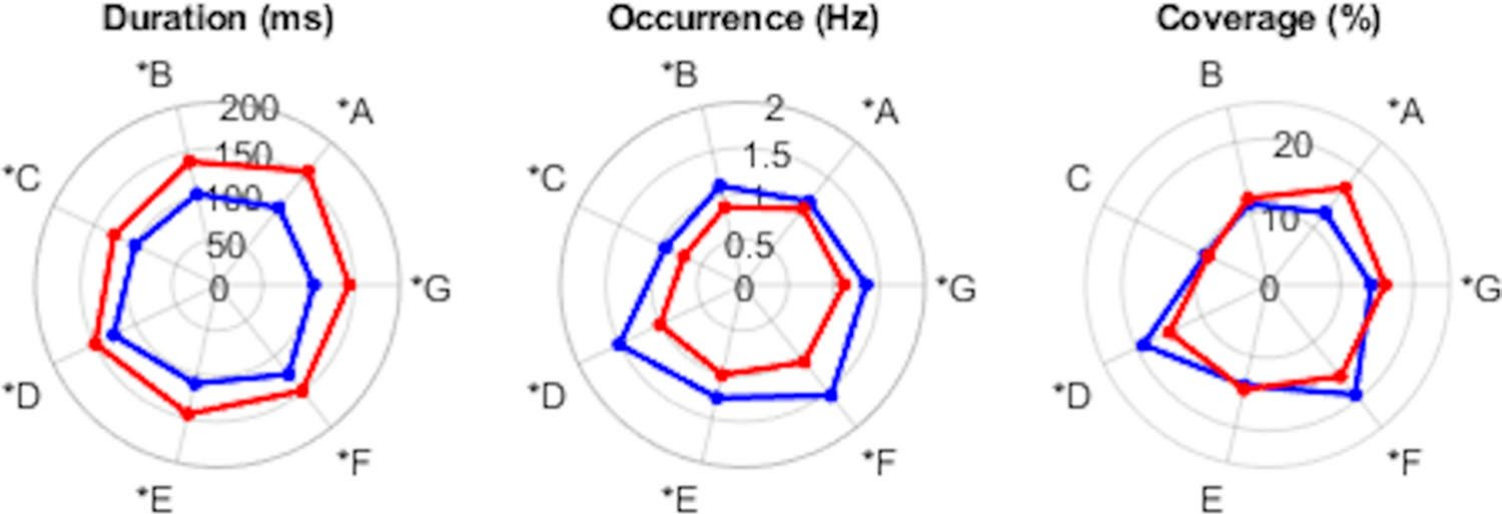
The average of microstate duration, occurrence, and coverage for all global microstate templates (A to G) extracted from broad-band EEG signals across AS (in blue) and QS (in red) epochs. Significant differences (p < 0.001) identified using post-hoc paired t-test are marked by an asterisk

The ANOVA on mean microstate duration and occurrence showed a significant main effect of *sleep states* (p < 0.001, Table 1)*.* In particular, the dominant microstate templates had a significantly longer duration in QS than in AS and occurred less frequently during QS than during AS (Fig. 4). A significant main effect of *microstate templates* was also found for all metrics (p < 0.001, Fig. 4), showing different duration, occurrence and coverage for the seven dominant microstate templates (Fig. 4, Table S2 in Online Resource 3), as well as a significant *sleep* *states* × *microstate templates* interaction (p < 0.001). The post hoc t-test showed significant differences (p < 0.001, Fig. 4, Table S2 in Online Resource 3) for the mean microstate duration (all dominant microstate templates), the mean microstate occurrence (all dominant microstate templates), and the mean microstate coverage (only dominant microstate templates A, D, F and G). Finally, significant differences were detected for the coverage of dominant microstate templates in QS and AS: microstate templates A and G had a higher coverage in QS than in AS, whereas microstate templates D and F had a higher coverage in AS than in QS. No significant differences were found for the coverage of the remaining microstate templates (Fig. 4).

**Table 1:** Results of Greenhouse–Geisser corrected repeated-measure ANOVA for mean microstate duration, occurrence and coverage

	*df*	*F*	*p*	$\eta _{p}^{2}$
Duration				
Sleep States	1.00, 59.00	254.03	^*^4.8E−23	0.812
Microstate templates	4.32, 254.99	27.54	^*^4.3E−20	0.318
Interaction	4.07, 239.96	15.17	^*^3.3E−11	0.205
Occurrence				
Sleep states	1.00, 59.00	218.37	^*^1.7E−21	0.787
Microstate templates	4.87, 287.35	71.43	^*^1.3E−47	0.548
Interaction	4.68, 276.07	24.64	^*^1.5E−19	0.295
Coverage				
Sleep states	NA	NA	NA	0
Microstate templates	4.64, 273.78	51.07	^*^1.8E−35	0.464
Interaction	4.58, 270.70	26.73	^*^1.2E−20	0.312

*NA* computation for group effect of coverage is not applicable because total coverage is 100%

*Statistically significant

**Fig. 5:**
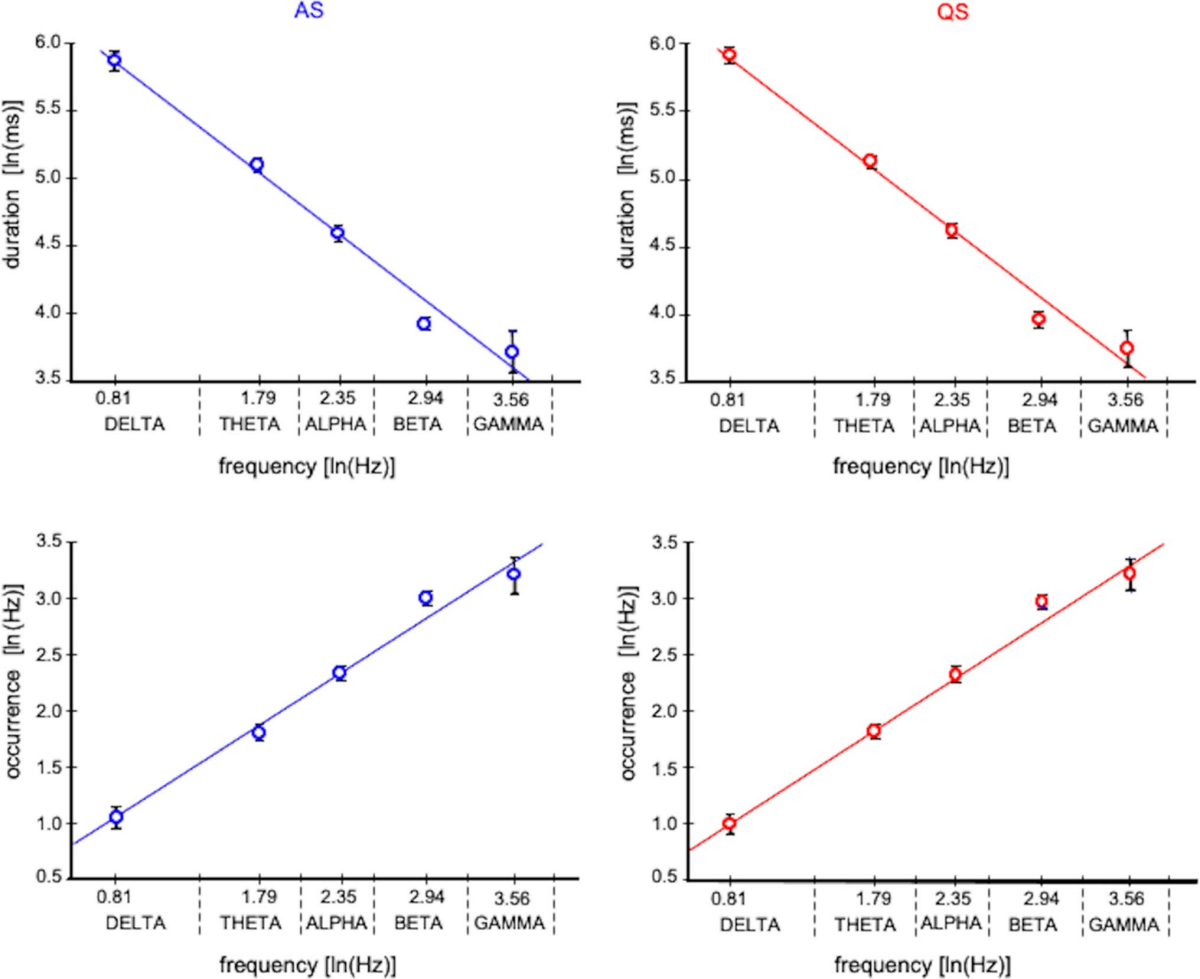
Log–log plot of mean values of duration and occurrence (averaged across all the seven templates) over the mean values of the delta, theta, alpha, beta and gamma frequency bands (natural logarithm of 2.25, 6, 10.5, 19, 35 Hz respectively) for both AS (blue) and QS (red). Vertical bars represent standard deviations

When considering the microstates extracted from the band filtered EEG data, a clear dependence of the microstate duration on frequency was found, so that microstates extracted from lower frequencies had a much longer duration (Fig. 5, Fig. S2 in Online Resource 4). In particular, the log–log plot of duration over frequency showed a negative linear relationship (slope of regression line -0.876 and -0.878 and R-squared value 0.986 and 0.968 for AS and QS respectively). Similarly, microstate occurrence changed with frequency, with an increase of occurrence values with higher frequencies (Fig. 5, Fig. S2 in Online Resource 4, slope of regression line 0.875 and 0.879 and R-squared value 0.986 and 0.989 for AS and QS respectively). A clear dependence of microstate coverage on frequency was not observed (Fig. S2 in Online Resource 4). A positive linear relationship between the log-transformed microstate duration and the log-transformed spectral power within each frequency band was found (Fig. S3 in Online Resource 5).

### Transitions between Microstates

**Fig. 6:**
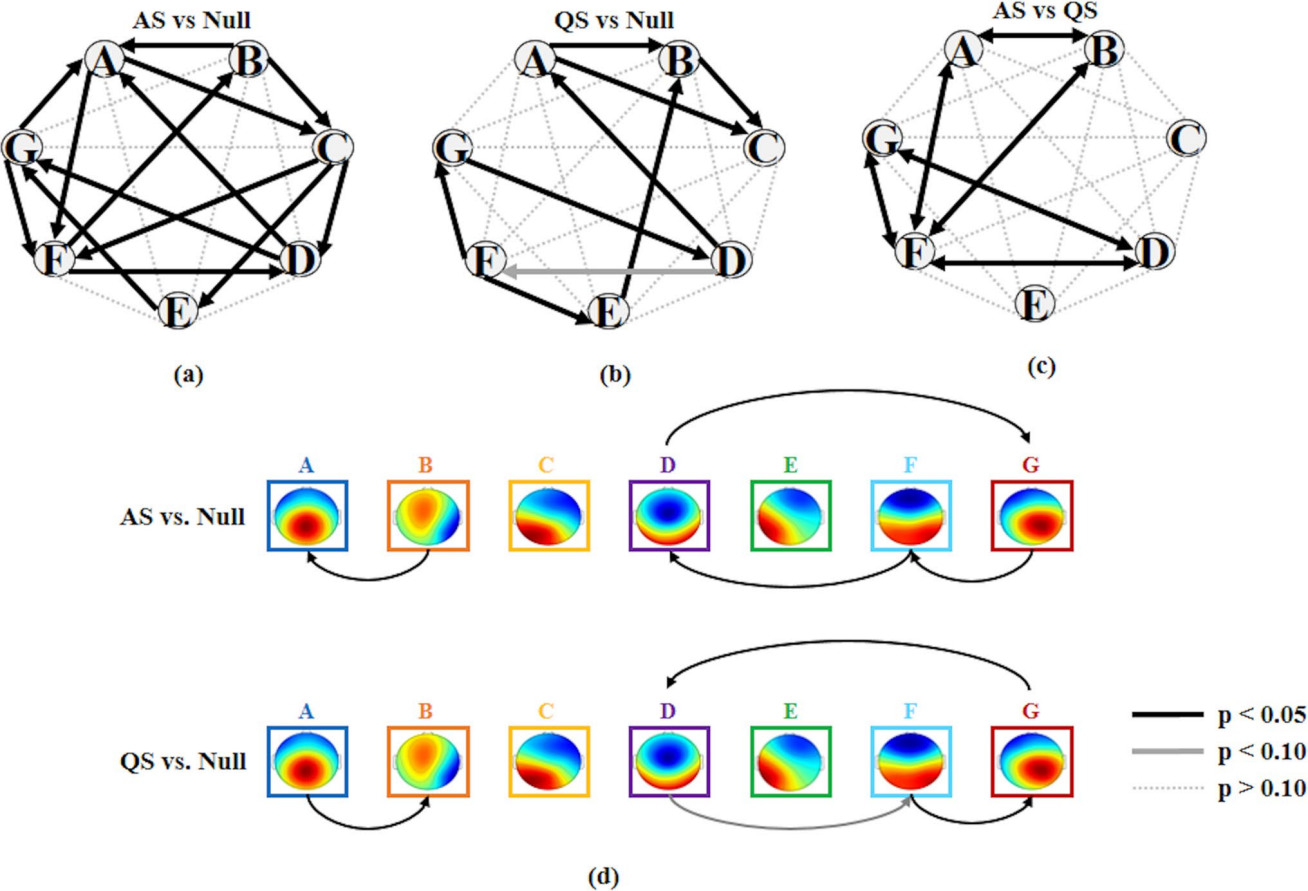
Microstate syntax in AS and QS states. **a**, **b** The directional predominance of transitions differed significantly from zero in AS and QS states (black and gray arrows). The directional transitions A → F, F → B, C → F and C → E were significant in AS and not significant in QS; the directional transition F → E was significant in QS and absent in AS; the transition loop A → F → B → A was significant in AS and absent in QS. **c** Transitions including A ↔ B, B ↔ F, D ↔ F, D ↔ G, F ↔ G showed significant differences in directional predominance. **d** Reversed transitions between microstates in AS and QS are given. The transition B → A was dominant in AS, whereas it was reversed in QS (A → B). Similarly, the transition loop D → G → F → D present in AS was reversed in QS (D → F → G → D)

The randomization test on the chi-squared distance between the *observed* and the *expected* transition probabilities in pairs of microstate templates showed, for both AS and QS, that the observed transitions followed a structure (AS: p = 0.0072; QS: p = 0.0084). In other words, the transitions from one microstate template to another during AS and QS were not determined by the occurrence frequencies of the microstate templates. For both AS and QS, the FDR corrected one-sample t-test showed significant positive and negative values of the directional predominance. This result indicates that significant preferential transitions between pairs of dominant microstate templates occurred in both sleep states (see Fig. 6a,b). The results of the repeated-measure ANOVA (2 *sleep states* × 21 *directional predominance* as within-subjects factors) showed a significant interaction between *sleep states* and *directional predominance* [F(10.36,611.67) = 6.080, p < 0.001,${\eta }_{p}^{2}=0.093$] and a significant main effect of *directional predominance* [F(9.97,588.71) = 4.98, p < 0.001,${\eta }_{p}^{2}=0.078$], whereas no significant main effect of *sleep states* (p > 0.300) was observed. The FDR corrected post hoc t-test between all possible pairs of directional predominance showed significant differences between specific microstate pairs in AS and QS states (Fig. 6c,d).

## Discussion

In this study, we identified seven microstates that characterize approximately 70% of the neonatal cortical activity during AS and QS states. This result is in agreement with adult studies in terms of the percentage of EEG variance that can be explained by the extracted microstate templates (Michel and Koenig 2018), although it must be observed that the optimal number of microstate templates for neonatal EEG—as determined with the Krzanowski-Lai (KL) criterion—was higher than in the case of adult EEG. The higher number of microstate templates required to reach high GEV in neonates as compared to adults could be due to the fact that newborn EEG is typically recorded and analyzed during sleep, whereas adult studies are typically performed during awake state. To our knowledge, only two microstate studies in adults reported data during non-REM sleep (no microstate studies on adult REM sleep exist), indicating lower GEV values in comparison with those obtained for newborns: Brodbeck et al. (2012) reported GEV between 60 and 67%, whereas Xu et al. (2020) reported GEV values around 65%.

When comparing the GEV values obtained in newborns with those obtained in adults, particular attention should be paid to the frequency content of EEG signals. In adult EEG, microstate analysis has been traditionally used to detect broad-band brain activity and dynamics (Michel and Koenig 2018) independently from the temporally overlapping sub-processes occurring at different frequencies that may contribute to the dynamics of the observable EEG signals. In most adult studies, EEG signals are bandpass-filtered between 1 (or 2) Hz and 30 (or 40) Hz, and it is well known that scalp EEG at rest in adults is dominated by alpha rhythm. Indeed, spatial fluctuations and temporal modulations of alpha power have been linked to the emergence and persistence of specific microstates (Milz et al. 2017; Croce et al. 2020). Nevertheless, recent studies raised the question about whether the broad-band activity should be spectrally differentiated in order to identify and describe spatio-temporally overlapping spectral patterns (Javed et al. 2019, 2020). A recent study in adults found that during NREM sleep low frequencies dominated in all microstates, whereas the EEG power for high frequencies was higher during wakefulness (Brechet et al. 2020). Our results confirmed that microstate analysis may be frequency-dependent, and the GEV is higher for lower oscillatory frequencies. This is compatible with the idea that neonatal EEG is dominated by lower frequencies (Vanhatalo et al. 2005) and that the spatial topography is more complex at higher frequencies, both in infants (Odabaee et al. 2013) and in adults (Freeman et al. 2003).

Our results showed that the global microstate templates are comparable between the two neonatal sleep states. Conversely, the EEG dynamics, described by the sequences of rapid transitions between microstate templates, is significantly different in AS and QS (Tokariev et al. 2016). In particular, we observed longer mean microstate duration and a reduced mean microstate occurrence during QS, which may relate to higher power associated with lower frequencies during QS vs. AS (Tokariev et al. 2016). The reduced microstate occurrence during QS can also be related to the increased microstate duration: the values of these two microstate metrics may indicate a generally lower information processing during QS, in agreement with adult findings on the underpinnings of deeper sleep states (Tononi and Massimini 2008).

The average microstate duration obtained from our cohort of neonatal EEG datasets was about 110–150 ms, much longer than the values found in adult microstate studies during different sleep states (Brodbeck et al. 2012), where an average microstate duration of about 40–100 ms was reported. These results seem to be in agreement with normative data on rest EEG in awake state that report a general dependence of microstate metrics on age. Koenig et al. reported that the average microstate duration was reduced from 6-years old children to 30-years old adults (children: 94.4 ± 3.4 ms; adults: 80.8 ± 3.4 ms), whereas the average microstate occurrence was increased (children:10.5 ± 0.4 Hz; adults: 12.4 ± 0.8 Hz) (Koenig et al. 2002). The prolonged average microstate duration observed in neonates could be related to the predominant lower frequency content of the infant EEG with respect to the adult EEG. Moreover, our data showed that microstate metrics depended on frequency. The log–log plot of duration over frequency showed a negative linear trend, suggesting a scale-invariant behavior of microstate dynamics in the neonatal EEG. Scale-invariant features of neonatal brain dynamics have been previously described (Iyer et al. 2015; Matic et al. 2015; Namazi and Jafari 2018), and, recently, Jannesari et al. (2020) observed, in the EEG of 6–12 month infants during sensory processing, suprathreshold events arranged in spatio-temporal clusters, whose size and duration follow a power-law (Jannesari et al. 2020). These events have been interpreted as hallmarks of neuronal *avalanches*, previously described in adults (Allegrini et al. 2010; Benayoun et al. 2010; Meisel et al. 2013; Palva et al. 2013; Priesemann et al. 2013; Shriki et al. 2013; Arviv et al. 2019). The stable topographies (microstates) whose duration follows a power-law, such as what we found for neonatal EEG during AS and QS, could be considered similar to the small-short local and wide-long global functional organization typical of *avalanches* (Petermann et al. 2009).

Our results also indicated that microstate sequences representing the EEG time course are non-casual. In other words, the co-activation of specific brain regions, resulting in a specific topographic map, facilitates the co-activation of another group of brain regions, in a sequential way that is compatible with time-parceled microstate sequences. We detected different non-casual directional transitions and transitional loops that were specific of QS and AS. This finding sides well with results of adult studies demonstrating that the transition probabilities between microstates are not casual, and that transition preferences occur (Lehmann et al., 2005) and are altered in pathological conditions (Nishida et al. 2013; Tomescu et al. 2015; Vellante et al. 2020). In adult studies, the sequences of microstates have been interpreted as an “evolutionary determined, brain-intrinsic biases toward particular patterns of co-activation particularly suited to represent environmental relevant information” (Michel and Koenig 2018). This interpretation can be adapted to EEG microstates in neonates. Our results showed quasi-stable patterns of spatio-temporal activity in the neonatal brain that were different in AS and QS. This phenomenological observation should be explained in terms of functional relevance and significance of microstate dynamics for the development of the neonatal brain. Future ad-hoc studies are needed to clarify this issue and to associate microstate features, dynamics and transitions with function and behavior.

In an attempt to understand the functional significance of microstates, combined EEG-fMRI studies in adults at rest associated each microstate with a specific resting-state network or an ensemble of resting-state networks (Britz et al. 2010; Michel and Koenig 2017; Rajkumar et al. 2020; Xu et al. 2020). These studies demonstrated that different microstate templates mirror distinct neuronal synchronized networks and that the microstate dynamics reflects the dynamic synchronization of such networks. The immaturity of the neurovascular coupling in neonates makes such direct interpolations across age groups problematic (Kozberg and Hillman 2016). However, recent EEG studies detected large-scale coupling in the neonatal neural activity, which seems to depend on the sleep state (Tokariev et al. 2016, 2019b; Tóth et al. 2017). Given that microstate templates represent the co-activation of neural pools in distinct brain areas and that their dynamics may characterize brain functions, an interesting question regards the relationship between functional connectivity features and EEG microstates in neonates. Future studies should investigate whether specific microstate templates may be associated with patterns of functional connectivity also in neonates, and whether the microstate features and sequences observed in AS and QS can be related to different developmental functions and behavior, as partially done in adults (Milz et al. 2016; Seitzman et al. 2017).

In conclusion, our results showed that: (1) the spatio-temporal dynamics contained in the neonatal EEG can be described by non-casual sequences of a limited number of dominant microstate templates; (2) the brain dynamics described by these microstate templates depends on frequency; (3) the features of the microstate sequences can capture and model the rapid changes occurring in the activity of the neonatal brain during different sleep states and can differentiate the different physiological conditions of AS and QS. Although further studies are needed to characterize the functional and developmental significance of microstate sequences, our study demonstrated that microstate analysis can contribute to advancing our knowledge of the mechanisms underpinning the onset and development of neural activity in the neonatal brain.

## Supplementary Information

Below is the link to the electronic supplementary material.

Supplementary file1 (PDF 278 kb)

Supplementary file2 (PDF 278 kb)

Supplementary file3 (PDF 289 kb)

Supplementary file4 (PDF 398 kb)

Supplementary file5 (PDF 346 kb)

## Data Availability

The data that support the findings of this study are available upon reasonable request.

## Code Availability

Data analysis was performed using the EEGLAB plugin developed by Thomas Koenig (www.thomaskoenig.ch/index.php/software/).
