# Supplementary material for: Characterization of the Functional Dynamics in the Neonatal Brain during REM and NREM Sleep States by means of Microstate Analysis
Source: Brain Topogr. 2021 Jul 13;34(5):555–67. doi: 10.1007/s10548-021-00861-1 (PMC8384814; doi:10.1007/s10548-021-00861-1)

Characterization of the Functional Dynamics in the Neonatal Brain during REM and NREM Sleep States by means of Microstate Analysis

Journal: Brain Topography

Mohammad Khazaei[1*], Khadijeh Raeisi[1*], Pierpaolo Croce[1], Gabriella Tamburro[1,2], Anton Tokariev[3,4,] Sampsa Vanhatalo[3,4], Filippo Zappasodi[1,5] , Silvia Comani[1,2]

[1] Department of Neuroscience, Imaging and Clinical Sciences, University "Gabriele d'Annunzio" of Chieti–Pescara, Chieti, Italy

[2] Behavioral Imaging and Neural Dynamics Center, University "Gabriele d'Annunzio" of Chieti–Pescara, Chieti, Italy

[3] BABA center, Pediatric Research Center, Department of Clinical Neurophysiology, Children's Hospital, Helsinki University Hospital and University of Helsinki, Helsinki, Finland

[4] Neuroscience center, Helsinki Institute of Life Science, University of Helsinki, Helsinki, Finland

[5] Institute for Advanced Biomedical Technologies, University "Gabriele d'Annunzio" of Chieti–Pescara, Chieti, Italy

[*]*Authors contributed equally to this work*.

Corresponding Author's Email: filippo.zappasodi@unich.it

Fig. S1 Mean across subjects and standard deviation of the Power Spectral Density (PSD) in Active Sleep (AS, blu) and Quiet Sleep (QS, red) states. For spectral estimation, a Welch method was used with a frequency resolution of 0.1 Hz.

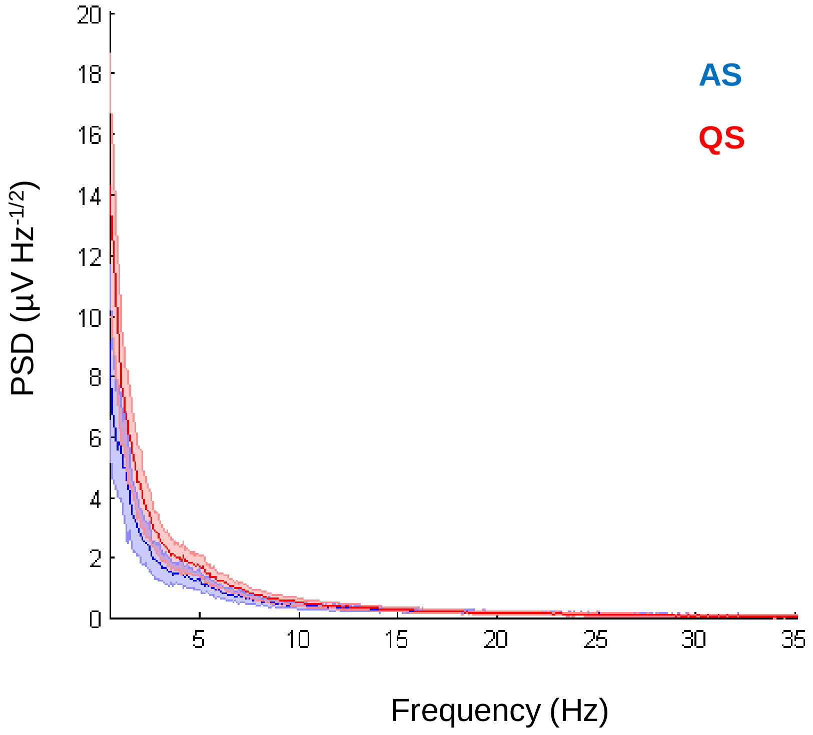

Supplement: Supplementary file 1 — Supplementary file1 (PDF 278 kb) [file 10548_2021_861_MOESM1_ESM.pdf]
