# Supplementary material for: Characterization of the Functional Dynamics in the Neonatal Brain during REM and NREM Sleep States by means of Microstate Analysis
Source: Brain Topogr. 2021 Jul 13;34(5):555–67. doi: 10.1007/s10548-021-00861-1 (PMC8384814; doi:10.1007/s10548-021-00861-1)
Supplement: Supplementary file 2 — Supplementary file2 (PDF 278 kb) [file 10548_2021_861_MOESM2_ESM.pdf]

Characterization of the Functional Dynamics in the Neonatal Brain during REM and NREM Sleep States by means of Microstate Analysis

Journal: Brain Topography

Mohammad Khazaei[1*], Khadijeh Raeisi[1*], Pierpaolo Croce[1], Gabriella Tamburro[1,2], Anton Tokariev[3,4,] Sampsa Vanhatalo[3,4], Filippo Zappasodi[1,5] , Silvia Comani[1,2]

[1] Department of Neuroscience, Imaging and Clinical Sciences, University "Gabriele d'Annunzio" of Chieti–Pescara, Chieti, Italy

[2] Behavioral Imaging and Neural Dynamics Center, University "Gabriele d'Annunzio" of Chieti–Pescara, Chieti, Italy

[3] BABA center, Pediatric Research Center, Department of Clinical Neurophysiology, Children's Hospital, Helsinki University Hospital and University of Helsinki, Helsinki, Finland

[4] Neuroscience center, Helsinki Institute of Life Science, University of Helsinki, Helsinki, Finland

[5] Institute for Advanced Biomedical Technologies, University "Gabriele d'Annunzio" of Chieti–Pescara, Chieti, Italy

[*] *Authors contributed equally to this work.*

Corresponding Author's Email: filippo.zappasodi@unich.it

Table S1 Mean ± standard deviation of the neonatal EEG power (expressed as ln(μV^2/Hz)) during AS and QS in the following frequency bands: delta (0.5-4 Hz), theta (4-8 Hz), alpha (8-13 Hz), beta (13-25 Hz), and gamma (25-45 Hz). T-values and p-values of the independent-sample t-test between AS and QS power for individual frequency bands are shown. Significant values are written in bold and marked by an asterisk (p < 0.001).

**Frequency band**	**Band Power AS**	**Band Power QS**	**T-value t(59)**	**p-value**
Delta	3.91±0.41	4.85±0.26	-18.05	***1.0E-25**
Theta	1.39±0.28	2.04±0.21	-16.81	***3.6E-24**
Alpha	-0.03±0.26	0.39±0.24	-13.36	***1.7E-19**
Beta	-0.31±0.33	-0.27±0.31	-0.80	0.429
Gamma	-1.19±0.62	-1.30±0.81	-1.18	0.241
