# Supplementary material for: Characterization of the Functional Dynamics in the Neonatal Brain during REM and NREM Sleep States by means of Microstate Analysis
Source: Brain Topogr. 2021 Jul 13;34(5):555–67. doi: 10.1007/s10548-021-00861-1 (PMC8384814; doi:10.1007/s10548-021-00861-1)
Supplement: Supplementary file 3 — Supplementary file3 (PDF 289 kb) [file 10548_2021_861_MOESM3_ESM.pdf]

Characterization of the Functional Dynamics in the Neonatal Brain during REM and NREM Sleep States by means of Microstate Analysis

Journal: Brain Topography

Mohammad Khazaei[1*], Khadijeh Raeisi[1*], Pierpaolo Croce[1], Gabriella Tamburro[1,2], Anton Tokariev[3,4,] Sampsa Vanhatalo[3,4], Filippo Zappasodi[1,5] , Silvia Comani[1,2]

[1] Department of Neuroscience, Imaging and Clinical Sciences, University “Gabriele d’Annunzio” of Chieti–Pescara, Chieti, Italy

[2] Behavioral Imaging and Neural Dynamics Center, University “Gabriele d’Annunzio” of Chieti–Pescara, Chieti, Italy

[3] BABA center, Pediatric Research Center, Department of Clinical Neurophysiology, Children’s Hospital, Helsinki University Hospital and University of Helsinki, Helsinki, Finland

[4] Neuroscience center, Helsinki Institute of Life Science, University of Helsinki, Helsinki, Finland

[5] Institute for Advanced Biomedical Technologies, University “Gabriele d’Annunzio” of Chieti–Pescara, Chieti, Italy

[*]*Authors contributed equally to this work*.

Corresponding Author’s Email: filippo.zappasodi@unich.it

Table S2 Results of the post hoc t-test for microstate metrics. Mean ± standard deviation of the microstate metrics are shown. Significant differences between AS and QS states, as assessed by independent sample t-test, are marked by an asterisk ($p < 0.001$). Computation of coverage is not applicable for "all microstates" because the total coverage is equal to 100 %.

	Templates	A	B	C	D	E	F	G	All microstates
Duration (ms)	***AS***	108.2±16.7	102.1±16.7	100.1±16.9	126.3±20.6	111.1±18.8	125.2±21.6	105.9±16.9	111.3 ± 20.7
	QS	159.9±24.4	138.7±36.2	126.4±19.6	148.6±26.8	145.5±21.4	148.5±18.8	145.2±23.1	144.7±26.5
	p	***3.2E-24**	***6.9E-12**	***6.2E-14**	***1.7E-8**	***4.3E-17**	***5.6E-10**	***1.8E-19**	----
Occurence (Hz)	***AS***	1.17±0.22	1.11±0.24	0.94±0.23	1.59±0.29	1.27±0.31	1.55±0.26	1.36±0.27	1.3 ± 0.3
	QS	1.06±0.19	0.87±0.16	0.72±0.02	1.00±0.21	1.01±0.20	1.08±0.18	1.12±0.18	1.0 ± 0.2
	p	***5.3E-4**	***1.8E-12**	***1.0E-8**	***8.9E-20**	***1.1E-8**	***4.4E-21**	***9.3E-10**	---
Coverage (%)	***AS***	12.7±2.8	11.4±3.0	9.5±3.0	18.9±4.5	14.1±3.9	19.2±3.5	14.2±2.8	---
	QS	17.0±4.3	12.1±4.3	9.1±2.8	14.8±3.9	14.7±3.7	16.0±2.9	16.2±3.5	---
	p	***1.7E-11**	0.178768	0.325474	***3.9E-8**	0.279867	***3.8E-9**	***2.3E-4**	---
