# Supplementary material for: Characterization of the Functional Dynamics in the Neonatal Brain during REM and NREM Sleep States by means of Microstate Analysis
Source: Brain Topogr. 2021 Jul 13;34(5):555–67. doi: 10.1007/s10548-021-00861-1 (PMC8384814; doi:10.1007/s10548-021-00861-1)

Characterization of the Functional Dynamics in the Neonatal Brain during REM and NREM Sleep States by means of Microstate Analysis

Journal: Brain Topography

Mohammad Khazaei[1*], Khadijeh Raeisi[1*], Pierpaolo Croce[1], Gabriella Tamburro[1,2], Anton Tokariev[3,4,] Sampsa Vanhatalo[3,4], Filippo Zappasodi[1,5], Silvia Comani[1,2]

[1] Department of Neuroscience, Imaging and Clinical Sciences, University "Gabriele d'Annunzio" of Chieti–Pescara, Chieti, Italy

[2] Behavioral Imaging and Neural Dynamics Center, University "Gabriele d'Annunzio" of Chieti–Pescara, Chieti, Italy

[3] BABA center, Pediatric Research Center, Department of Clinical Neurophysiology, Children's Hospital, Helsinki University Hospital and University of Helsinki, Helsinki, Finland

[4] Neuroscience center, Helsinki Institute of Life Science, University of Helsinki, Helsinki, Finland

[5] Institute for Advanced Biomedical Technologies, University "Gabriele d'Annunzio" of Chieti–Pescara, Chieti, Italy

[*]*Authors contributed equally to this work.*

Corresponding Author's Email: filippo.zappasodi@unich.it

Fig. S2 Mean ± standard deviation of microstate duration, occurrence and coverage are shown for each microstate extracted from the band-pass filtered EEG signals in the delta (δ: 0.5-4 Hz), theta (θ: 4-8 Hz), alpha (α: 8-13 Hz), beta (β: 13-25 Hz), and gamma (γ: 25-45 Hz) frequency bands for AS (blue) and QS (red).

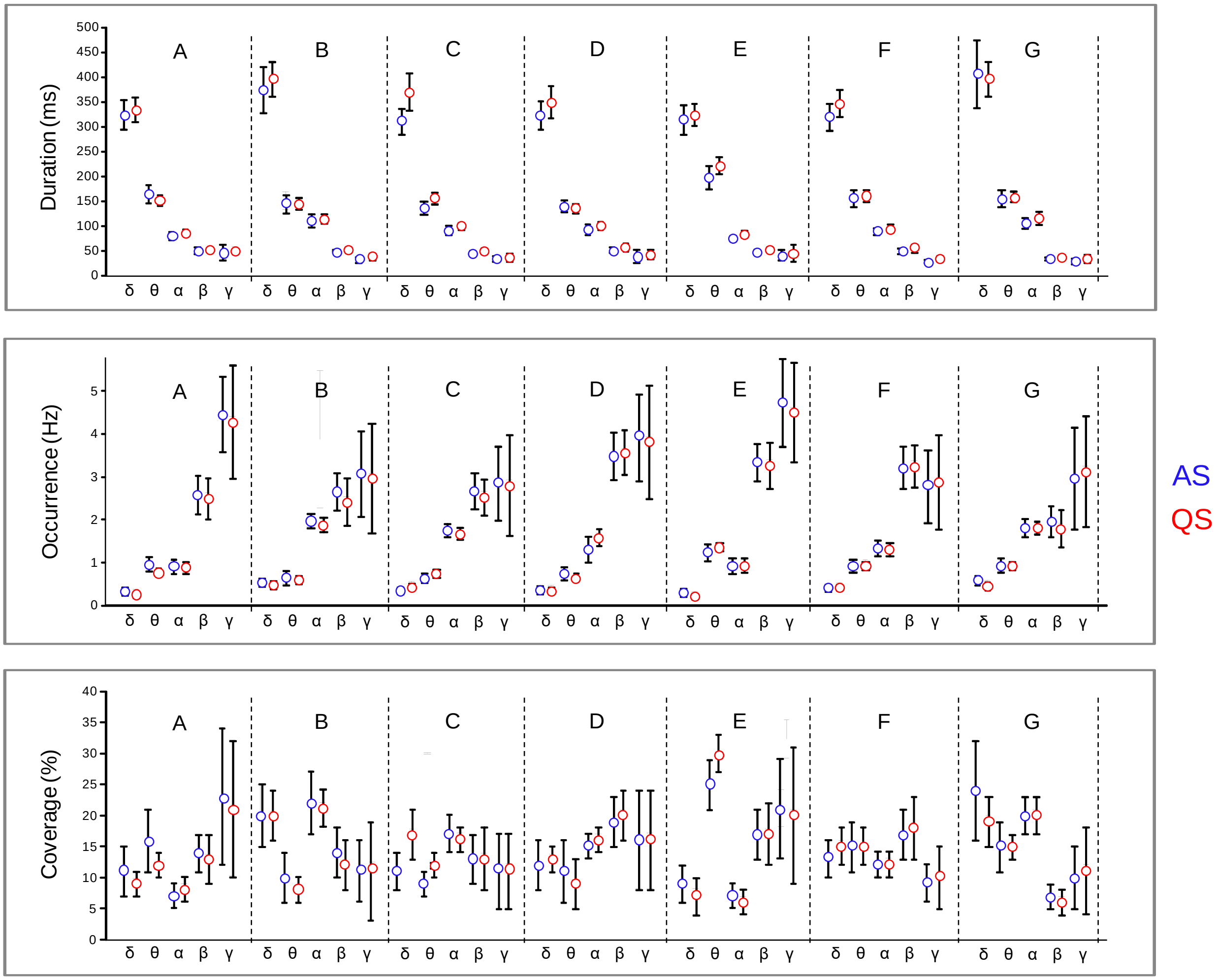

Supplement: Supplementary file 4 — Supplementary file4 (PDF 398 kb) [file 10548_2021_861_MOESM4_ESM.pdf]
