# Supplementary material for: Characterization of the Functional Dynamics in the Neonatal Brain during REM and NREM Sleep States by means of Microstate Analysis
Source: Brain Topogr. 2021 Jul 13;34(5):555–67. doi: 10.1007/s10548-021-00861-1 (PMC8384814; doi:10.1007/s10548-021-00861-1)

Characterization of the Functional Dynamics in the Neonatal Brain during REM and NREM Sleep States by means of Microstate Analysis

Journal: Brain Topography

Mohammad Khazaei[1*], Khadijeh Raeisi[1*], Pierpaolo Croce[1], Gabriella Tamburro[1,2], Anton Tokariev[3,4,] Sampsa Vanhatalo[3,4], Filippo Zappasodi[1,5] , Silvia Comani[1,2]

[1] Department of Neuroscience, Imaging and Clinical Sciences, University "Gabriele d'Annunzio" of Chieti–Pescara, Chieti, Italy

[2] Behavioral Imaging and Neural Dynamics Center, University "Gabriele d'Annunzio" of Chieti–Pescara, Chieti, Italy

[3] BABA center, Pediatric Research Center, Department of Clinical Neurophysiology, Children's Hospital, Helsinki University Hospital and University of Helsinki, Helsinki, Finland

[4] Neuroscience center, Helsinki Institute of Life Science, University of Helsinki, Helsinki, Finland

[5] Institute for Advanced Biomedical Technologies, University "Gabriele d'Annunzio" of Chieti–Pescara, Chieti, Italy

[*]*Authors contributed equally to this work*.

Corresponding Author's Email: filippo.zappasodi@unich.it

Fig. S3 Log-log plot of mean values of the duration over the mean values of the spectral power in the delta, theta, alpha, beta and gamma frequency bands for both AS (blue) and QS (red). Vertical bars represent standard deviations. A positive linear relationship can be observed (slope of regression line 0.429 and 0.370 and R-squared value 0.921 and 0.959 for AS and QS respectively).

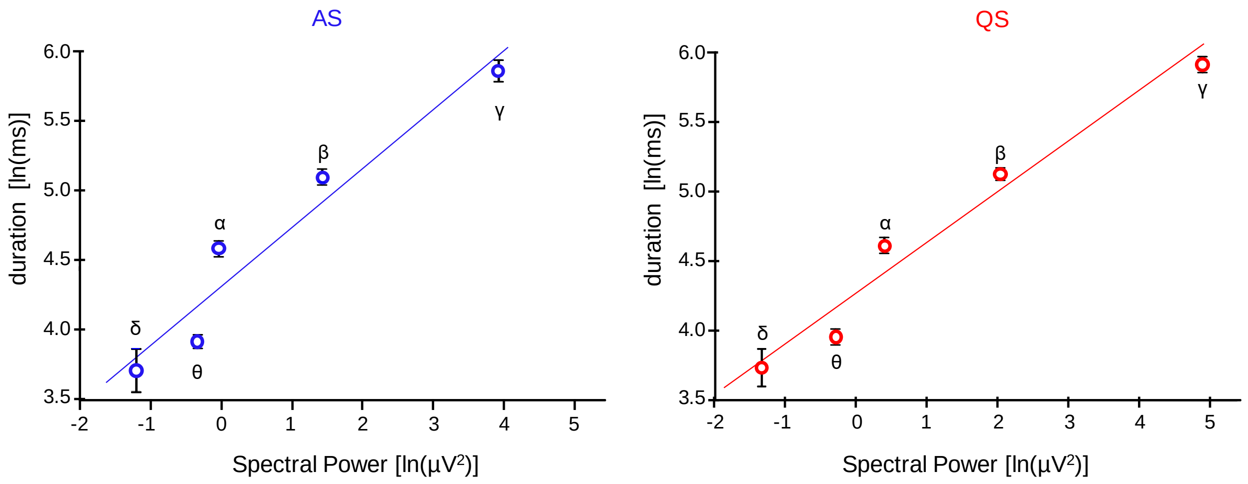

Supplement: Supplementary file 5 — Supplementary file5 (PDF 346 kb) [file 10548_2021_861_MOESM5_ESM.pdf]
